# Hydrogel Encapsulating Wormwood Essential Oil with Broad‐spectrum Antibacterial and Immunomodulatory Properties for Infected Diabetic Wound Healing

**DOI:** 10.1002/advs.202305078

**Published:** 2023-11-29

**Authors:** Feng Wang, Qi Sun, Yang Li, Ruijun Xu, Renjie Li, Dingcai Wu, Rongkang Huang, Zifeng Yang, Yong Li

**Affiliations:** ^1^ Guangdong Cardiovascular Institute Guangdong Provincial People's Hospital Guangdong Academy of Medical Sciences Guangzhou 510080 China; ^2^ Department of Gastrointestinal Surgery Department of General Surgery Guangdong Provincial People's Hospital (Guangdong Academy of Medical Sciences) Southern Medical University Guangzhou 510080 China; ^3^ School of Medicine South China University of Technology Guangzhou 510006 China; ^4^ PCFM Lab School of Chemistry Sun Yat‐sen University Guangzhou 510006 China; ^5^ The Eighth Affiliated Hospital Sun Yat‐sen University Shenzhen 518033 China; ^6^ Department of General Surgery (Colorectal Surgery) Guangdong Institute of Gastroenterology Biomedical Innovation Center Guangdong Provincial Key Laboratory of Colorectal and Pelvic Floor Diseases The Sixth Affiliated Hospital Sun Yat‐sen University Guangzhou 510655 China; ^7^ Key Laboratory of Biowaste Resources for Selenium‐Enriched Functional Utilization, College of Petroleum and Chemical Engineering Beibu Gulf University Qinzhou 535011 China

**Keywords:** antibacterial, hydrogel dressing, immunomodulatory, wormwood essential oil, wound healing

## Abstract

The integration of hydrogels with bio‐friendly functional components through simple and efficient strategies to construct wound dressings with broad‐spectrum antibacterial and immunomodulatory properties to promote the healing of infected diabetic wounds is highly desirable but remains a major challenge. Here, wormwood essential oil (WEO) is effectively encapsulated in the hydrogel via an O/W‐Pickering emulsion during the polymerization of methacrylic anhydride gelatin (GelMA), acrylamide (AM), and acrylic acid *N*‐hydroxysuccinimide ester (AAc‐NHS) to form a multifunctional hydrogel dressing (HD‐WEO). Compared with conventional emulsions, Pickering emulsions not only improve the encapsulation stability of the WEO, but also enhance the tensile and swelling properties of hydrogel. The synergistic interaction of WEO's diverse bioactive components provides a broad‐spectrum antibacterial activity against *S. aureus*, *E. coli*, and *MRSA*. In addition, the HD‐WEO can induce the polarization of macrophages from M1 to M2 phenotype. With these advantages, the broad‐spectrum antibacterial and immunomodulatory HD‐WEO effectively promotes the collagen deposition and neovascularization, thereby accelerating the healing of *MRSA*‐infected diabetic wounds.

## Graphical abstract

Wormwood essential oil (WEO) is effectively encapsulated in the hydrogel via an O/W‐Pickering emulsion during the polymerization of methacrylic anhydride gelatin (GelMA), acrylamide (AM), and acrylic acid *N*‐hydroxysuccinimide ester (AAc‐NHS) to form a multifunctional hydrogel dressing (HD‐WEO). The HD‐WEO exhibits excellent broad‐spectrum antibacterial activity and intrinsic immunomodulatory property for the healing of infected diabetic wounds.

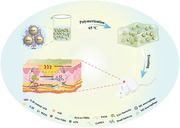

## 1 Introduction

The increasing prevalence of diabetes has led to the gradual emergence of infected wounds as a significant public health problem.^[^ ^1^ ^]^ Effective wound healing requires both antibacterial treatment and regulation of the immune microenvironment.^[^ ^2^ ^]^ However, the presence of hyperglycemia in infected diabetic wound could impair its immune function, leading to a propensity for macrophages to adopt the M1 phenotype rather than the M2 phenotype. As a result, this change in phenotype could lead to an upregulation of proinflammatory cytokines expression and stimulation of oxidative stress.^[^ ^3^ ^]^ These unfavorable factors ultimately hinder the wound healing process and increase the likelihood of severe secondary complications, including cellulitis, abscess formation, and sepsis. Therefore, dressings that possess excellent antibacterial and adequate immunomodulatory properties play a critical role in the effective management of infected diabetic wounds.^[^ ^4^ ^]^

Conventional wound dressings, such as gauze, foam, and gelatin sponge have the ability to absorb exudate or maintain moisture. However, their inadequacy in terms of antibacterial and immunomodulatory properties renders them incapable of treating the infected diabetic wounds.^[^ ^5^ ^]^ In recent years, hydrogel dressings can be designed to resist bacteria and absorb exudate for infected diabetic wounds.^[^ ^6^ ^]^ However, the efficacy and safety of some conventional antibacterial hydrogel dressings is often hindered by their antibacterial agents (Table S1, Supporting Information). For example, antibiotics possess a narrow spectrum and can easily lead to the emergence of multidrug‐resistant pathogens. The utilization of metals or metal oxides increases the risk of potential heavy metal toxicity associated with long‐term use.^[^ ^7^ ^]^ The application of photothermal antibiotics may cause thermal damage to wound tissue,^[^ ^8^ ^]^ and photodynamic antibiotics may induce oxidative stress in wounds.^[^ ^9^ ^]^ In addition, some of these antibacterial agents have a low antimicrobial ratio (Table S1, Supporting Information). Poor infection control and oxidative stress have a certain negative impact on the local immune microenvironment of wounds.^[^ ^10^ ^]^ Therefore, it is of great significance to develop a kind of high‐performance hydrogel dressing with both antibacterial and immunomodulatory properties to promote the healing of infected diabetic wounds.

With the development of phytochemistry, essential oils have been used as antibacterial and immunomodulatory agents due to the synergistic effect of diverse bioactive components.^[^ ^11^ ^]^ However, the hydrophobicity of essential oils makes them difficult to integrate with hydrophilic hydrogel dressings, and the volatility of essential oils hinders their efficient penetration into biofilms containing highly charged extracellular polymers,^[^ ^11a^ ^]^ thereby limiting their efficacy in the treatment of infected diabetic wounds. It has been demonstrated that the encapsulation of essential oils in colloidal delivery systems, facilitated by surfactants, can enhance the payloads and water stability of essential oils.^[^ ^12^ ^]^ However, most surfactants, especially anionic and cationic surfactants,^[^ ^13^ ^]^ have a tendency to dissolve lipid membranes and induce skin irritation reactions, including adverse hemolysis and functional disorders, thereby limiting their biocompatibility.^[^ ^14^ ^]^ Meanwhile, surfactant stabilized emulsions exhibit inadequate thermal stability, making it difficult to maintain stability during thermal polymerization. Therefore, how to efficiently integrate hydrophobic essential oil and hydrophilic hydrogel together to build a wound dressing with broad‐spectrum antibacterial activity, adequate immunoregulatory properties, and good biocompatibility is an important scientific issue to promote healing of infected diabetic wounds.

**Figure 1:**
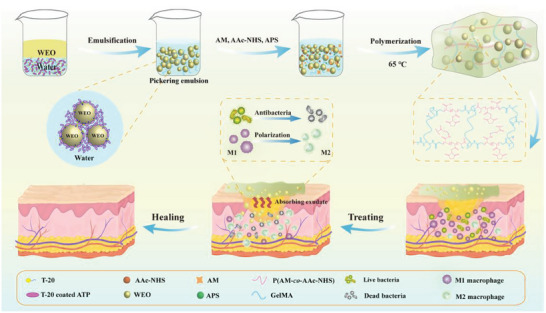
The preparation process diagram of HD‐WEO and the mechanism to promote healing of infected diabetic wound.

In this study, Pickering emulsion and free radical polymerization were used to integrate natural wormwood essential oil (WEO) with biocompatible hydrogel to construct a class of wound dressing with antibacterial activity, immunomodulatory property, and good biocompatibility (**Figure** **1**). Biocompatible attapulgite (ATP) nanoclay and a small amount of edible Tween‐20 (T‐20) were used to synergistically stabilize the WEO‐in‐water Pickering emulsion.^[^ ^11^, ^15^ ^]^ Methacrylic anhydride gelatin (GelMA), acrylamide (AM), and acrylic acid *N*‐hydroxysuccinimide ester (AAc‐NHS) in the continuous phase of the emulsion were thermally initiated by free radical polymerization to construct the hydrogel matrix. Due to the excellent anti‐agglomeration and stability of the Pickering emulsion,^[^ ^16^ ^]^ WEO is effectively encapsulated in the hydrogel during polymerization to form multifunctional hydrogel dressing (HD‐WEO). Compared with conventional emulsions, Pickering emulsions not only improve the encapsulation stability of the WEO, but also enhance the tensile properties of the HD‐WEO. The WEO contributes to the broad‐spectrum antibacterial activity and immunomodulatory property of HD‐WEO.^[^ ^17^ ^]^ The 3D network structure of HD‐WEO ensures good tensile and swelling performances, enabling effective coverage of various irregular wounds and absorption of exudate.^[^ ^18^ ^]^ In addition, HD‐WEO contains abundant hydroxyl, amino, and succinimide ester groups, which promote adhesion to the skin and facilitate its clinical use.^[^ ^19^ ^]^ With the above advantages, our HD‐WEO can effectively promote the healing of *MRSA*‐infected diabetic wounds on rats, demonstrating its promising application for the treatment of infected diabetic wounds.

## 2 Results and Discussion

**Figure 2:**
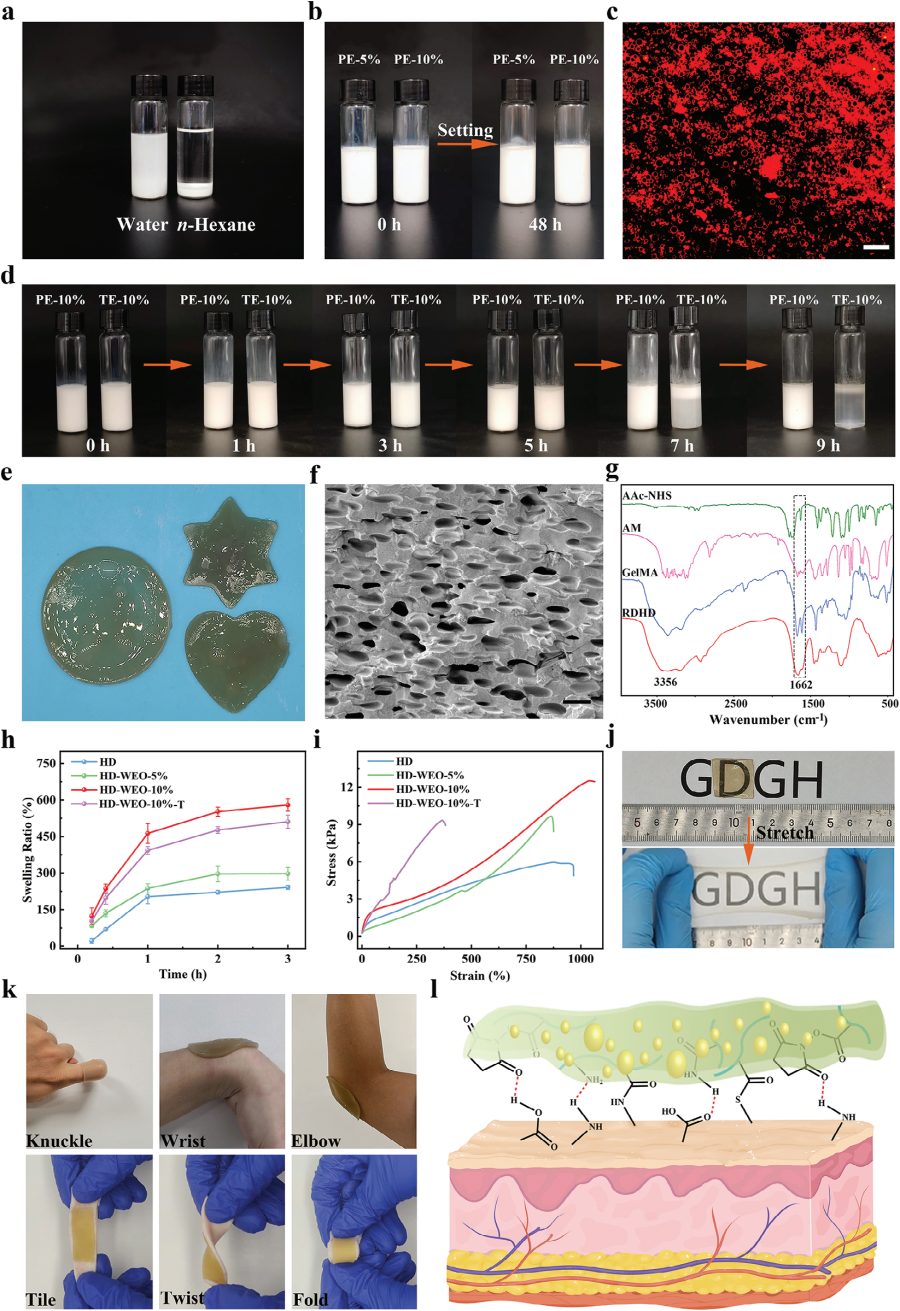
a) Testing the type of PE‐10% by dropwise adding the emulsion into the water and *n*‐hexane. b) Digital photos of freshly prepared PE‐5% and PE‐10% and those were stored for 48 h. c) CLSM image of PE‐10% (ATP was stained with safranine T). Scale bar: 50 µm. d) Digital photos of PE‐10% and TE‐10% after being stored at 65 °C at different times. e) Digital photo of HD‐WEO hydrogels in various shapes. f) SEM image of RDHD (HD‐WEO‐10% after oil removal and drying). Scale bar: 5 µm. g) FT‐IR spectra of AAc‐NHS, AM, GelMA, and RDHD. h) Swelling ratios of HD and HD‐WEOs (The error bars show a standard deviation, *n* = 3). i) Tensile strength of HD and HD‐WEOs. j) Digital photos of HD‐WEO‐10% before and after stretching. k) Digital photos of HD‐WEO‐10% adhered to moving joints and porcine skin. l) The adhesion principle of HD‐WEOs to the skin.

We elaborately select ATP and T‐20 with good hydrophilicity and biosafety as stabilizers to construct WEO‐in‐water Pickering emulsions (PE‐*x*, *x* representing the WEO phase mass fraction). The T‐20 is coated on the ATP by hydrogen bonding to regulate the wettability of the ATP, making it more effective in stabilizing the oil‐water interface.^[^ ^16b^ ^]^ The droplet test shows that the emulsion exhibits good dispersion in the water but forms aggregates in the *n*‐hexane, suggesting that the emulsion type is oil‐in‐water (**Figure** **2**a). After 48 h of storage, the emulsions remain stable without noticeable phase separation, indicating good stability (Figure 2b). The confocal laser scanning microscope (CLSM) image of PE‐10% in Figure 2c shows that ATP (stained with safranine T) is present both at the oil‐water interface and in the continuous phase. ATP forms a 3D network and contributes to emulsion stability by preventing droplet coalescence and creaming. The diffusion of ATP in the continuous phase is caused by the inherent high surface hydrophilicity of ATP and T‐20, which can form a strong hydrated layer, causing intramolecular cohesion and intermolecular steric hindrance.^[^ ^20^ ^]^ In addition, our Pickering emulsion (PE‐10%) exhibits superior thermal stability than the emulsion stabilized with T‐20 alone (TE‐10%), ensuring effective encapsulation of WEO during thermal polymerization (Figure 2d). The results suggest that WEO can be effectively encapsulated in the Pickering emulsion stabilized with ATP and T‐20.

Figure 1 shows a schematic of HD‐WEO preparation process. Briefly, ATP and T‐20 were first dispersed in GelMA solution.^[^ ^31^ ^]^ WEO was then added to the suspension at different mass fractions (5% and 10%) and the mixtures were vigorously stirred to form Pickering emulsions. After the emulsion formation, AM, AAc‐NHS, and APS were added to the emulsion solution to undergo free radical polymerization and ultimately produce HD‐WEO. The resulting HD‐WEO could be easily shaped using a mold or cut (Figure 2e) to fit different geometries of infected diabetic wounds. During polymerization, P(AM‐*co*‐AAc‐NHS) is formed by the copolymerization of AM and AAc‐NHS and cross‐linked by GelMA through C—C, amide, and hydrogen bonds. SEM image of the RDHD (HD‐WEO‐10% after removing oil and drying) shows a typical emulsion‐templated pore structure (Figure 2f) with pore diameters ranging from 1.0 to 5.3 µm (Figure S1, Supporting Information), resulting from the removal of the oil phase. As shown in the FT‐IR spectrum (Figure 2g) of RDHD, the peak at 3356 cm^−1^ is derived from O─H and N─H stretching vibrations of GelMA and PAM segment;^[^ ^22^ ^]^ the peak at 1662 cm^−1^ is derived from the C═O stretching vibration of the PAM segment, GelMA,^[^ ^4^ ^]^ and PAAc‐NHS segment.^[^ ^23^ ^]^ Additionally, we also prepared a hydrogel dressing without WEO, named HD, as well as an HD‐WEO prepared solely with T‐20 stabilized emulsion, named HD‐WEO‐10%‐T.

A high swelling ratio is important for dressing to effectively absorb exudate. We evaluated the swelling ratios of HD, HD‐WEO‐5%, HD‐WEO‐10%, and HD‐WEO‐10%‐T using PBS. HD, HD‐WEO‐5%, and HD‐WEO‐10% exhibit swelling ratios of ≈2.4, 3.0, and 5.8 times their own weight, respectively (Figure 2h). The increase in swelling ratio can be attributed to the decrease in water content of HD, HD‐WEO‐5%, and HD‐WEO‐10%.^[^ ^24^ ^]^ Moreover, commercial dressings such as Hydrosorb and DuoDERM dressings demonstrate the swelling ratios of only ≈two times their own weights. Notably, HD‐WEO‐10% displays a higher swelling ratio (Figure 2h) and better anti‐reflux effect (Figure S2, Supporting Information) than the HD‐WEO‐10%‐T. This might be attributed to the fact that ATP enhances the network structure of the HD‐WEO‐10%, resulting in improved water absorption and retention properties. These results show that HD‐WEO‐10% is highly effective in absorbing exudate.

The HD‐WEO possesses favorable tensile properties due to its physical and chemical crosslinking network. Figure 2i illustrates the remarkable tensile strength and elongation of the HD, HD‐WEO‐5%, HD‐WEO‐10%, and HD‐WEO‐10%‐T. Interestingly, the tensile strength of HD, HD‐WEO‐5%, and HD‐WEO‐10% exhibits a progressive increase as the water content decreases. This is because the decrease in water content enhances the entanglement of the polymer chains, eventually leading to the increased tensile properties of the hydrogel dressings.^[^ ^25^ ^]^ The elongation values of HD‐WEO range from 865% to 1049%, exceed those of several other hydrogel dressings, which typically exhibit elongation values below 100%.^[^ ^26^ ^]^ In addition, the elongation value and tensile strength of HD‐WEO‐10 % are both higher than that of HD‐WEO‐10%‐T, which may be attributed to the potential role of ATP in enhancing hydrogel's cross‐linking degree, thus strengthening and toughening the HD‐WEO‐10%. Our HD‐WEO‐10% can be stretched to ≈ten times its initial length (Figure S3, Supporting Information), and is capable of stretching in all directions (Figure 2j). Due to the excellent tensile strength, our HD‐WEO could meet the full coverage requirements for irregular infected diabetic wounds.

Appropriate tissue adhesion and good tensile properties of wound dressings are essential to effectively and conveniently cover irregular wounds, thus reducing the risk of infection.^[^ ^27^ ^]^ Our HD‐WEO‐10% can adhere firmly to dynamic human skin surfaces such as elbow, wrist, interphalangeal joints, and fresh pigskin without retraction or tearing under bending or stretching conditions (Figure 2k). Figure S4a (Supporting Information) further demonstrates that HD‐WEO‐10% could conform to human knuckles at various angles without wrinkling. When the hydrogel is peeled off, sticky fibrils form at the interface between the HD‐WEO‐10% and the skin, indicating a high degree of attachment (Figure S4b, Supporting Information). Furthermore, no hydrogel residue or allergic reaction is observed on the skin even after 24 h of adhesion, confirming the good biocompatibility of the HD‐WEO‐10% (Figure S4c, Supporting Information).^[^ ^28^ ^]^ The tissue adhesive properties of HD‐WEO‐10% are attributed to the presence of abundant —OH, —NH_2_, and NHS ester groups, enabling interactions with —NH_2_ and ‐SH present in tissue peptides and proteins (Figure 2l).^[^ ^29^ ^]^ When the HD‐WEO‐10% is in contact with the skin tissue surface, physical crosslinking is rapidly formed between ─OH and —NH_2_ groups in the HD‐WEO‐10% and those in biological tissues.^[^ ^29^, ^30^ ^]^ Covalent cross‐linking is further formed between NHS ester groups in HD‐WEO‐10% and primary amine and sulfydryl groups in biological tissues, ensuring long‐term adhesion stability.^[^ ^29^, ^30^ ^]^ Additionally, HD‐WEO‐10% also shows strong adhesion to other substrates such as steel, glass, and plastic (Figure S4d, Supporting Information). These results indicate that our HD‐WEO possesses sufficient adhesion to human skin, meeting the requirement of dressing for good tissue adhesion in clinical use.

**Figure 3:**
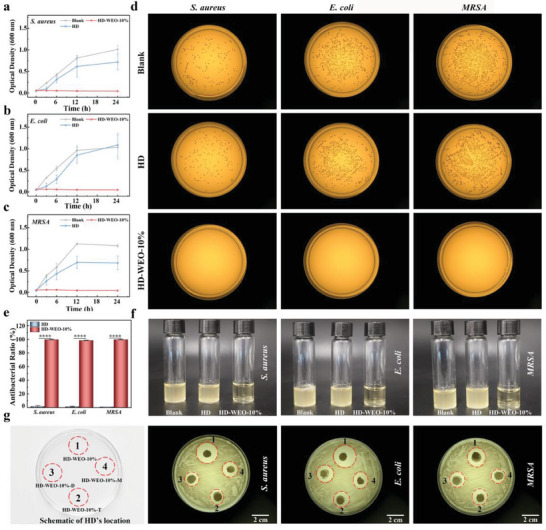
a–c) Growth curves of *S. aureus* (a)*, E. coli* (b), and *MRSA* (c) were evaluated as a function of culture time in the blank, HD, and HD‐WEO‐10% groups. d) Digital photos of the viable bacterial clones formed by *S. aureus, E. coli*, and *MRSA* on agar plates for 24 h after contacting with HD and HD‐WEO‐10%. e) Antibacterial ratios of the blank, HD, and HD‐WEO‐10% groups for *S. aureus, E. coli*, and *MRSA*. f) Digital photos of *S. aureus, E. coli*, and *MRSA* solutions cocultured with HD and HD‐WEO‐10% after 24 h. g) Digital photos of survival *S. aureus*, *E. coli*, and *MRSA* clones on agar plates after contacting with HD‐WEO‐10%, HD‐WEO‐10%‐T, HD‐WEO‐10%‐D, and HD‐WEO‐10%‐M. The red circles indicate bacterial clones that were killed by HD‐WEO. The error bars show a standard deviation (*n* = 3).

One of the major challenges in infected diabetic wound healing is bacterial infection.^[^ ^31^ ^]^ Methicillin‐resistant *Staphylococcus aureus* (*MRSA*) has been detected in >90% of diabetic wounds due to frequent and inappropriate use of antibiotics in clinic.^[^ ^7^, ^32^ ^]^ However, the current research on diabetic wound dressings for the treatment of *MRSA* could be insufficient (Table S2, Supporting Information). Therefore, there is a need for wound dressings that effectively inhibit drug‐resistant bacteria without inducing further resistance in diabetic wounds. WEO contains several antibacterial compounds such as 1,8‐cineole, 1‐caryophyllene, camphor, α‐terpineol, eugenol, and germacrene D. These compounds work synergistically to exert antibacterial properties by targeting multiple pathways rather than a single one, thus reducing the likelihood of developing drug‐resistance bacteria.^[^ ^33^ ^]^ We evaluated the antibacterial efficacy of the HD and HD‐WEO‐10% against three common wound pathogens (*S. aureus*, *E. coli*, and *MRSA*) and compared them with the blank group.^[^ ^34^ ^]^ **Figure** **3**a–c illustrates that the optical density (OD) values of *S. aureus*, *E. coli*, and *MRSA* in both the blank and HD groups exhibit a notable increase initially, reaching their peak at 12 h. Conversely, the OD value in HD‐WEO‐10% group shows no increase within 24 h.^[^ ^28^ ^]^ The results of agar plate experiments show that HD‐WEO‐10% achieves ≈100% killing efficiency for all three types of bacteria, which is superior to the HD and blank groups (Figure 3d,e). Furthermore, the culture solution of HD‐WEO‐10% group appears transparent after 24 h, suggesting minimal bacterial growth, while the solutions of the HD and blank groups appear turbid, indicating substantial bacterial growth (Figure 3f). We also performed an antibacterial ring test to investigate the releasable bactericidal properties of the HD‐WEO‐10%. Three control samples (HD‐WEO‐10%‐T, HD‐WEO‐10%‐D, and HD‐WEO‐10%‐M) were employed for control experiments (Figure 3g). HD‐WEO‐10%‐D refers to HD with WEO droplets present on the surface, while HD‐WEO‐10%‐M is obtained by mixing the WEO into the hydrogel precursor prior to polymerization. The results of the antibacterial ring test show that our HD‐WEO‐10% has the largest inhibition ring (Figure 3g), demonstrating that WEO can be effectively encapsulated in HD by Pickering emulsion to improve its antibacterial properties.

**Figure 4:**
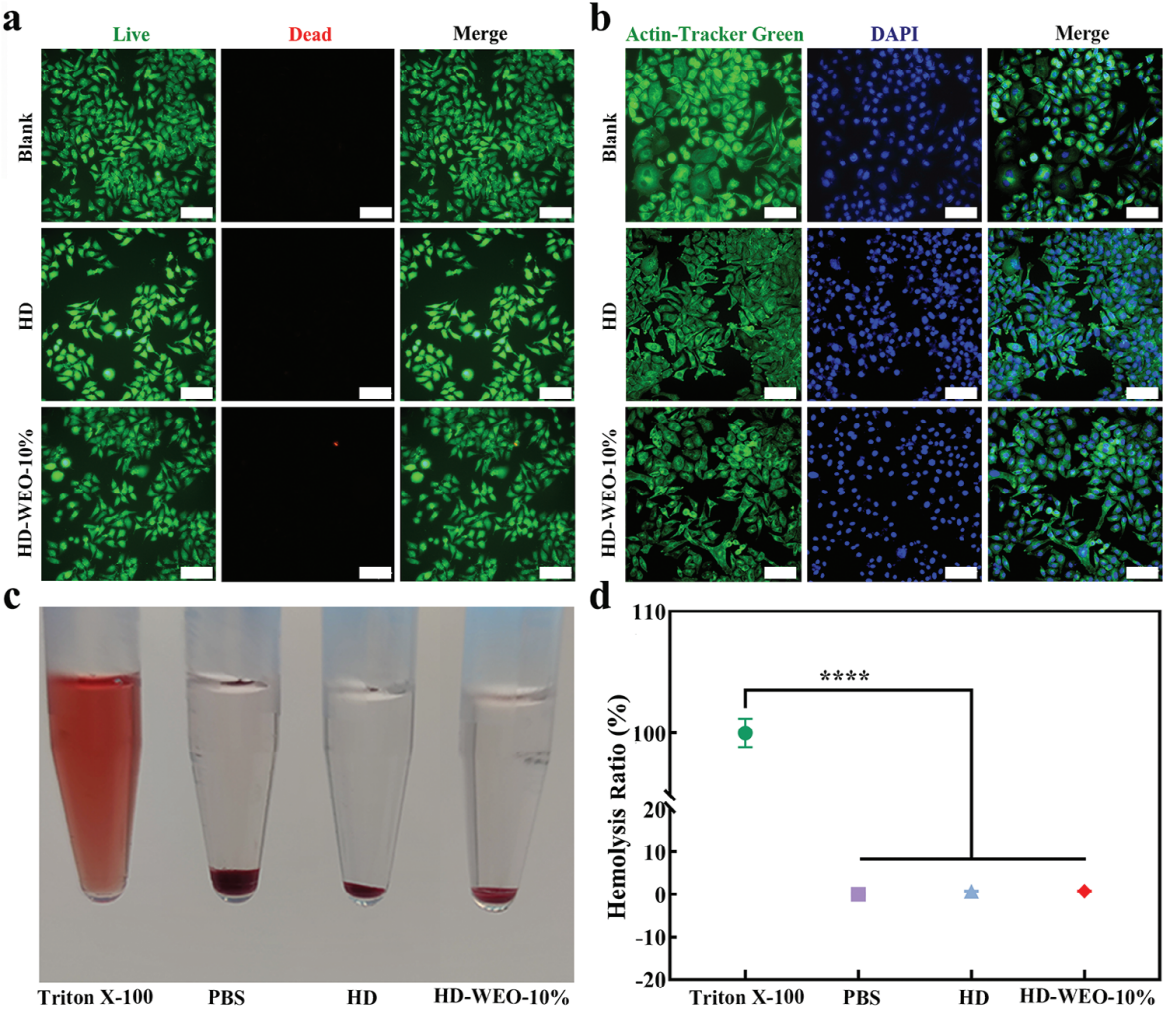
a) Microscopic images of a live/dead assay of L929 cells from the blank, HD, and HD‐WEO‐10% groups. Scale bar: 100 µm. b) Microscopic images of cytoskeleton staining of L929 cells from the blank, HD, and HD‐WEO‐10% groups. Scale bar: 100 µm. c) Digital photos of hemolytic activity assay of the Triton‐100, PBS, HD, and HD‐WEO‐10% groups. d) The hemolysis ratios of the Triton‐100, PBS, HD, and HD‐WEO‐10% groups. The error bars show a standard deviation (*n* = 3). ^****^ *p* <0.0001.

Good biocompatibility is an essential characteristic for clinical wound dressings. To investigate the in vitro cytotoxicity of HD‐WEO‐10%, we performed a leaching assay using L929 cells.^[^ ^35^ ^]^ L929 cells were exposed to extraction solutions of HD and HD‐WEO‐10% for 24 h, followed by staining with CCK‐8 to assess cell viability. The results indicate that the cell viability of HD and HD‐WEO‐10% exceeds 90% (Figure S5, Supporting Information). The live/dead cell assay in **Figure** **4**a shows that L929 cells in the HD and HD‐WEO‐10% groups exhibit a spindle‐like morphology with green fluorescence, while few dead cells exhibit red fluorescence after incubation. As shown in Figure 4b, L929 cells in all groups exhibit normal cytoskeletal (green) and nuclear (blue) morphology. The cells are densely arranged in a spindle‐like pattern, demonstrating good biocompatibility of HD‐WEO‐10%.^[^ ^28^ ^]^

An ideal wound dressing should demonstrate minimal or no hemolysis when in contact with bleeding wounds.^[^ ^27^ ^]^ The hemocompatibility of HD‐WEO was assessed by an in vitro hemolysis assay. HD and HD‐WEO‐10% were co‐incubated with blood at 37 °C for 24 h, and the resulting supernatants were obtained after centrifugation. Figure 4c illustrates that the supernatants from the HD and HD‐WEO‐10% groups exhibit a nearly colorless, with sedimented red blood cells at the bottom, similar to the negative PBS control group. In contrast, the positive Triton X‐100 group displays a vivid red coloration. The hemolysis rates of HD and HD‐WEO‐10% are close to 0% (Figure 4d). These results confirm that our HD‐WEO‐10% does not induce hemolysis.^[^ ^36^ ^]^

In order to assess the histocompatibility of HD‐WEO‐10%, a subcutaneous embedding experiment was conducted on rats, with a commercial gelatin sponge (GS) as the control group. The results indicate that there is no tissue swelling or any other adverse reactions observed in the GS and HD‐WEO‐10% groups (Figure S6a, Supporting Information). The quantitative analysis of inflammatory factors (CD68, IL‐1*β*, and IL‐6) reveals no significant differences between the two groups (Figure S6b,c, Supporting Information). This indicates that our HD‐WEO‐10% exhibits good tissue compatibility, which is comparable to that of commercial GS.

**Figure 5:**
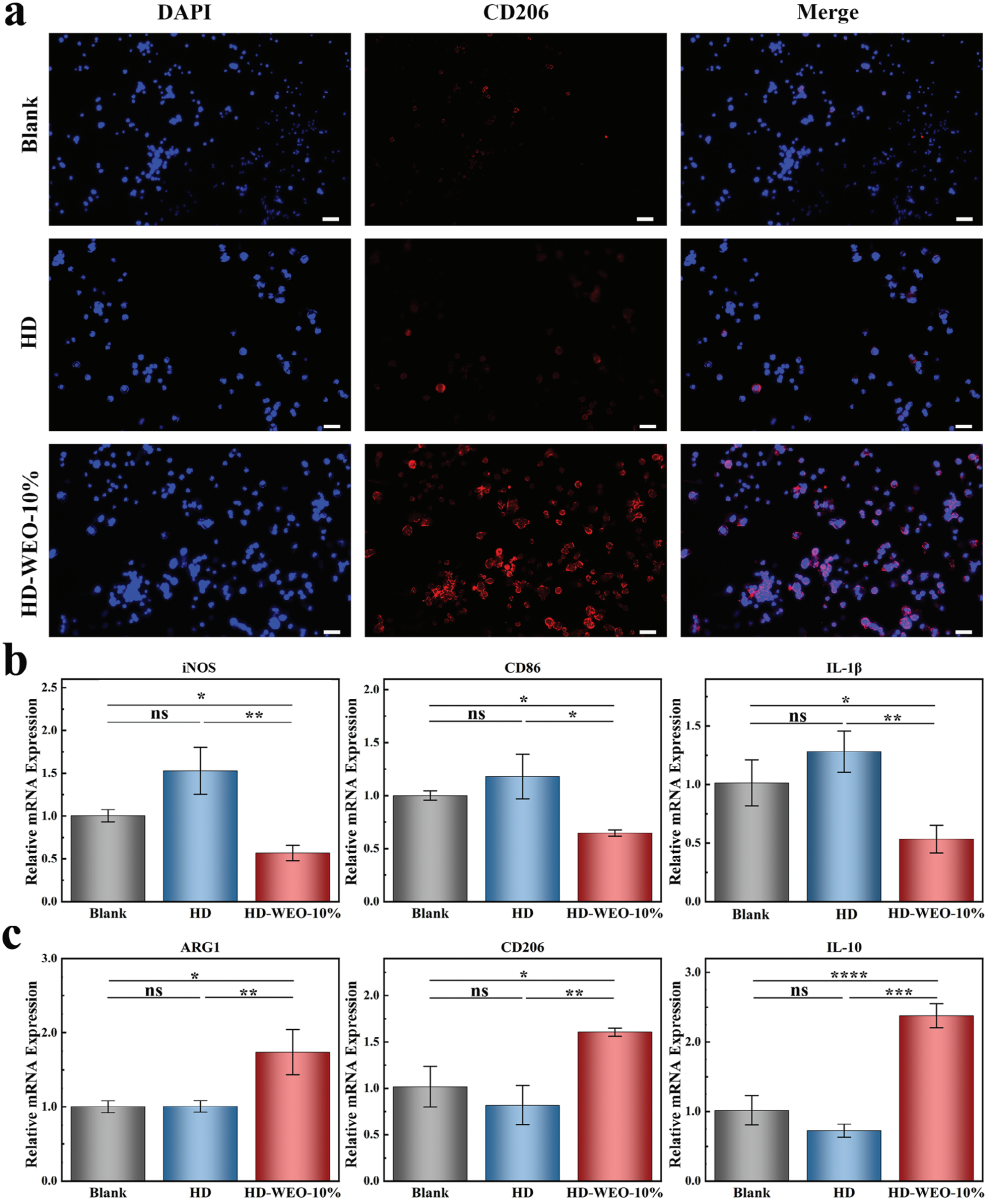
a) Microscopic images of a cluster of differentiation CD206 (red) in Raw264.7 cells from the blank, HD, and HD‐WEO‐10% groups. Scale bar: 100 µm. b, c) Relative mRNA levels of M1 (b) and M2 (c) markers in macrophages from the blank, HD, and HD‐WEO‐10% groups. The error bars show a standard deviation (*n* = 3). ns: not significant, ^*^ *p* < 0.05, ^**^ *p* < 0.01, ^***^ *p* < 0.001, and ^****^ *p* < 0.0001.

Macrophages, a type of innate immune cell, play a crucial role in wound healing and immune regulation.^[^ ^37^ ^]^ The presence of phenolic and flavonoid compounds in WEO gives it immunomodulatory properties.^[^ ^38^ ^]^ Therefore, we investigated the ability of HD‐WEO‐10% to induce macrophages polarization from M1 to M2 phenotype in vitro. The immunofluorescence staining reveals that the expression of CD206, a marker of M2 macrophages,^[^ ^3b^ ^]^ is significantly higher in HD‐WEO‐10% group compared to HD group (**Figure** **5**a). Specifically, the proportion of CD206 positive cells is found to be 9.6% for HD and 77.6% for HD‐WEO‐10% (Figure S7, Supporting Information). According to the results of RT‐PCR, the expression levels of M2 markers (CD206, IL‐10, and ARG‐1) are significantly upregulated in macrophages co‐cultured with HD‐WEO‐10%. Conversely, the expression levels of M1 markers (CD86, IL‐1β, and iNOS) exhibit a significant decrease (Figure 5b). These results demonstrate that HD‐WEO‐10% effectively induces the polarization of macrophages from M1 to M2 phenotype. This process leads to the suppression of pro‐inflammatory mediators and the enhancement of anti‐inflammatory mediators, thereby promoting diabetic wound healing.^[^ ^39^ ^]^ The anti‐inflammatory effects of WEO may be attributed to its inhibition of JAK/STATs signaling and scavenging of reactive oxygen species, resulting in reduced protein and mRNA expression levels of inflammatory factors.^[^ ^17a^ ^]^

**Figure 6:**
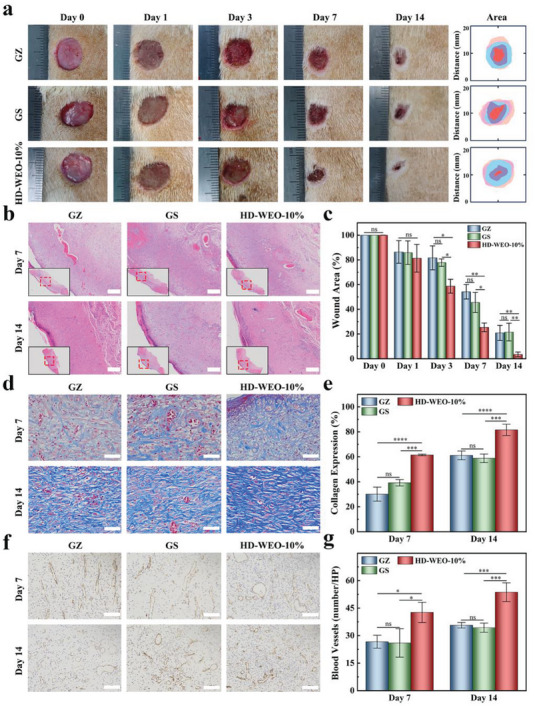
a) Digital photos of the wounds in the GZ, GS, and HD‐WEO‐10% groups. b) H&E staining images of wounds in the GZ, GS, and HD‐WEO‐10% groups on the 7th and 14th days. Scale bar: 250 µm. c) Statistical analysis of the wound areas. d) Masson staining images of wounds in the GZ, GS, and HD‐WEO‐10% groups on the 7th and 14th days. Scale bar: 50 µm. e) Statistical analysis of collagen expressions. f) CD31 staining images of wounds in the GZ, GS, and HD‐WEO‐10% groups on the 7th and 14th days. Scale bar: 100 µm. g) Statistical analysis of the number of blood vessels. The error bars show a standard deviation (*n* = 3). ns: not significant, ^*^ *p* < 0.05, ^**^ *p* < 0.01, ^***^ *p* < 0.001, and ^****^ *p* < 0.0001.

The promising results observed in vitro have motivated us to conduct further investigation the therapeutic effectiveness of HD‐WEO‐10% in a more complex in vivo setting. We established an *MRSA*‐infected diabetic wound model and administered HD‐WEO‐10% to the infected wound, comparing it with commercial gauze (GZ), and gelatin sponge (GS) (Figure S8, Supporting Information). The progression of wound healing was monitored, and tissue samples were collected on 0th, 3rd, 7th, and 14th days. The results indicate that HD‐WEO‐10% significantly improves wound closure compared to GZ and GS (**Figure** **6**a), with healing rates of 75% and 95% on 7th and 14th days, respectively (Figure 6c). Histological analysis using H&E staining reveals that the histological characteristics are consistent with the rate of wound healing (Figure 6b). The HD‐WEO‐10% group shows faster wound contraction than the other groups. On the 14th day, HD‐WEO‐10% exhibits dense granulation tissue, regenerated epidermis, and dermis, achieving the highest degree of area closure (Figure 6b). These results suggest that our HD‐WEO‐10% is particularly effective in promoting wound healing.

Adequate deposition and remodeling of collagen is essential for enhancing tissue strength and promoting expedited healing.^[^ ^1^ ^]^ Masson's staining was employed to assess the newly formed collagen (Figure 6d). The HD‐WEO‐10% group exhibits elevated levels of collagen expression compared to the GZ and GS groups. Notably, the HD‐WEO‐10% group demonstrates a peak collagen expression of ≈81.6% on day 14, which is significantly higher than the GZ (61.2%) and GS (58.8%) groups (Figure 6e). Neovascularization plays a crucial role in wound healing, as it facilitates the transport of oxygen, nutrients, and immune cells to the injured site, thereby promoting the proliferation of skin‐related cells, synthesis of collagen, and re‐epithelialization.^[^ ^40^ ^]^ To evaluate new blood vessels in the granulation tissue, immunostaining for platelet‐endothelial cell adhesion molecule (CD31) was conducted on the 7th and 14th days. Figure 6f shows that the HD‐WEO‐10% group exhibits higher levels of CD31 than the GZ and GS groups. The average number of new blood vessels in the GZ, GS, and HD‐WEO‐10% groups is ≈27, 26, and 43 per high‐power field (HP) on the 7th day, and ≈36, 34, and 54 per HP on the 14th day (Figure 6g). These results demonstrate that our HD‐WEO‐10% can effectively promote collagen deposition and accelerate neovascularization in *MRSA*‐infected diabetic wounds.

**Figure 7:**
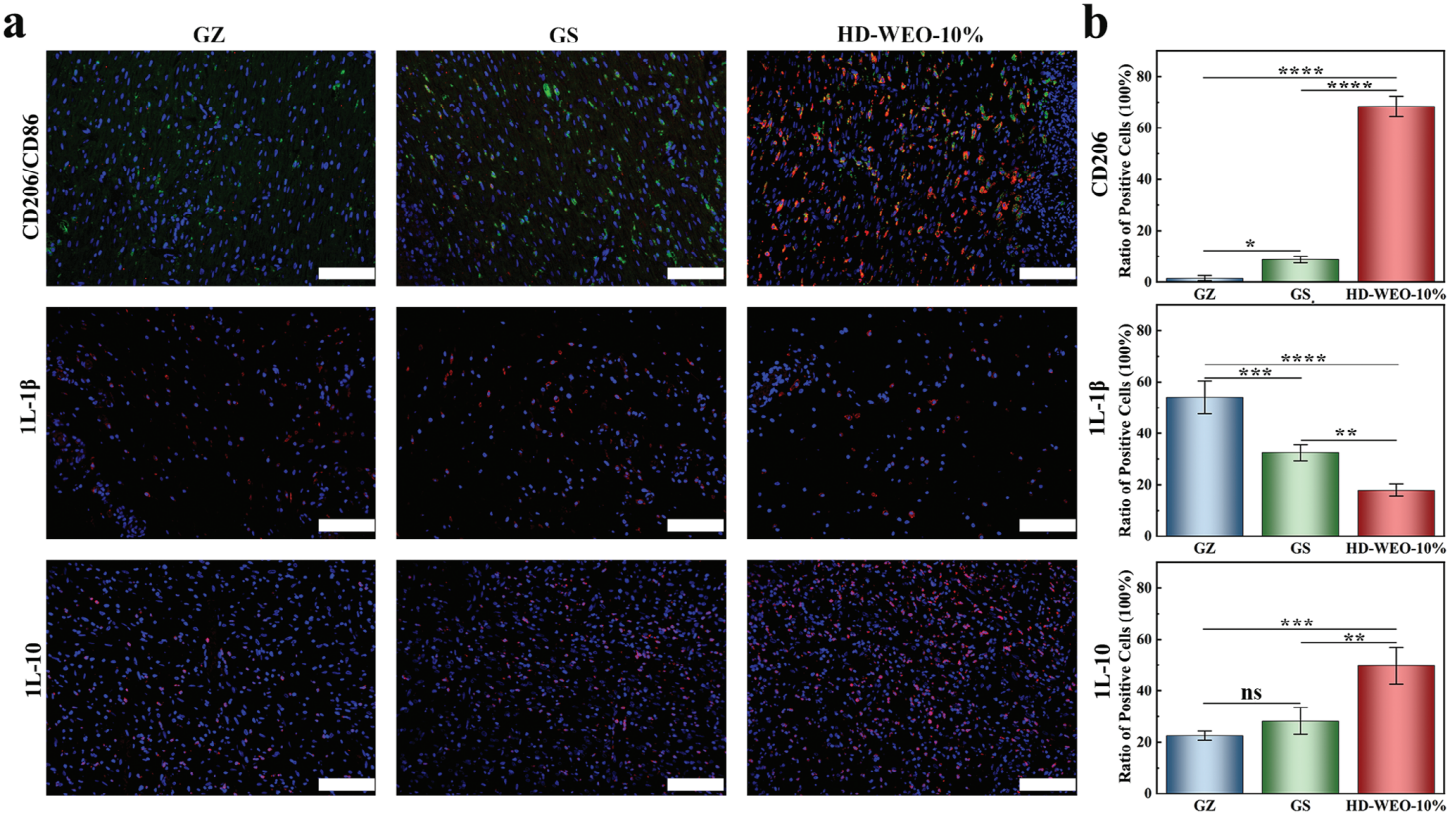
a) Immunofluorescence staining images of M2 marker gene (CD206), M1 cytokine (IL‐1β), and M2 cytokine (IL‐10) in the GZ, GS, and HD‐WEO‐10% groups on the 7th day. b) The rate of CD206, IL‐1β, and IL‐10 positive cells per field in the GZ, GS, and HD‐WEO‐10% groups. The error bars show a standard deviation (*n* = 3). ns: not significant, ^*^ *p* < 0.05, ^**^ *p* < 0.01, ^***^ *p* < 0.001, and ^****^ *p* < 0.0001.

Our HD‐WEO has demonstrated the ability to promote macrophage polarization toward M2 in vitro. To further investigate the correlation between the wound healing and the polarization status of macrophages in diabetic wounds, we collected the tissue samples from the wounds treated with different dressings on the 7th day post‐injury and performed immunohistochemistry staining. As shown in **Figure** **7**a, HD‐WEO‐10% group exhibits a significantly higher presence of M2 macrophages (CD206) than GZ and GS groups. Immunofluorescence staining was used to analyze the protein levels of M1 cytokines (IL‐1β) and M2 cytokines (IL‐10). HD‐WEO‐10% group exhibits lower protein levels of M1 cytokines and higher levels of M2 cytokines (Figure 7a,b). These results indicate that HD‐WEO‐10% may promote macrophage polarization toward M2 in vivo to enhance the healing process.

## 3 Conclusion

A promising HD‐WEO has been successfully developed, demonstrating activity against drug‐resistant bacteria and intrinsic immunoregulatory properties for the healing of infected diabetic wounds. Through a bio‐friendly Pickering emulsion, the hydrophobic bio‐functional WEO is effectively encapsulated into the hydrophilic hydrogel to address the issue of WEO volatility. The copolymerization of GelMA, AM, and AAc‐NHS not only ensures good stretchability and swelling ratio of the HD‐WEO, but also enhances the adhesion of the hydrogel to the skin tissue by hydrogen bonding and covalent cross‐linking between numerous functional groups in the HD‐WEO and the skin tissue. Consequently, HD‐WEO provides full coverage and self‐adhesion on various irregular wounds. The as‐obtained HD‐WEO exhibits excellent biocompatibility, antibacterial property, and can modulate the immune microenvironment of diabetic wound infected with drug‐resistant bacteria. It stimulates macrophage polarize toward the M2 phenotype without the exogenous addition of expensive biological factors. As a result, our HD‐WEO effectively accelerates the healing of infected diabetic wound and shows great potential as a novel type of wound dressing.

## 4 Experimental Section

All materials and methods can be found in the accompanying supporting information.

## Conflict of Interest

The authors declare no conflict of interest.

## Supporting information

Supporting Information

## Data Availability

The data that support the findings of this study are available in the supplementary material of this article.
