# Supplementary material for: Hydrogel Encapsulating Wormwood Essential Oil with Broad‐spectrum Antibacterial and Immunomodulatory Properties for Infected Diabetic Wound Healing
Source: Adv Sci (Weinh). 2023 Nov 29;11(3):2305078. doi: 10.1002/advs.202305078 (PMC10797468; doi:10.1002/advs.202305078)
Supplement: Supplementary file 1 — Supporting Information [file ADVS-11-2305078-s001.pdf]

ADVANCED SCIENCE
Open Access

Supporting Information

for *Adv. Sci.*, DOI 10.1002/advs.202305078

Hydrogel Encapsulating Wormwood Essential Oil with Broad-spectrum Antibacterial and Immunomodulatory Properties for Infected Diabetic Wound Healing

Feng Wang, Qi Sun, Yang Li, Ruijun Xu, Renjie Li, Dingcai Wu, Rongkang Huang*, Zifeng Yang* and Yong Li**

Supporting Information

Hydrogel Encapsulating Wormwood Essential Oil with Broad-spectrum Antibacterial and Immunomodulatory Properties for Infected Diabetic Wound Healing

Feng Wang, Qi Sun, Yang Li, Ruijun Xu, Renjie Li, Dingcai Wu*, Rongkang Huang*, Zifeng Yang*, Yong Li*

Dr. F. Wang, Y. Li, Dr. R. Li, Prof. Y. Li

Guangdong Cardiovascular Institute

Guangdong Provincial People's Hospital

Guangdong Academy of Medical Sciences

Guangzhou, 510080, China

E-mail: liyong@gdph.org.cn

Dr. F. Wang, Y. Li, Dr. R. Li, Dr. Z. Yang, Prof. Y. Li

Department of Gastrointestinal Surgery, Department of General Surgery

Guangdong Provincial People's Hospital (Guangdong Academy of Medical Sciences)

Southern Medical University

Guangzhou, 510080, China

E-mail: yangzifeng@gdph.org.cn

Q. Sun, R. Xu, Prof. Y. Li

School of Medicine

South China University of Technology

Guangzhou, 510006, China

Prof. D. Wu

PCFM Lab

School of Chemistry

Sun Yat-sen University

Guangzhou 510006, China

E-mail: wudc@mail.sysu.edu.cn

Prof. D. Wu

The Eighth Affiliated Hospital

Sun Yat-sen University

Shenzhen 518033, China

Dr. R. Huang

Department of General Surgery (Colorectal Surgery)

Guangdong Institute of Gastroenterology

Biomedical Innovation Center

Guangdong Provincial Key Laboratory of Colorectal and Pelvic Floor Diseases

The Sixth Affiliated Hospital

Sun Yat-sen University

Guangzhou 510655, China

E-mail: huangrk3@mail.sysu.edu.cn

Dr. F. Wang

Key Laboratory of Biowaste Resources for Selenium-Enriched Functional Utilization

College of Petroleum and Chemical Engineering

Beibu Gulf University

Qinzhou 535011, China

Methods and Materials

1. Materials

Methacrylylated gelatin (GelMA) was purchased from Engineering for Life Co., Ltd. (China). Wormwood essential oil (WEO), acrylamide (AM), acrylic acid *N*-hydroxysuccinimide ester (AAc-NHS), ammonium persulfate (APS) and Tween-20 (T-20) were purchased from Shanghai Macklin Biochemical Technology Co., Ltd. (China). ATP was provided by Lanzhou Institute of Chemical Physics, Chinese Academy of Sciences. The commercial gauze was purchased from Cofoe Medical Technology Co., Ltd. (China), and the commercial gelatin sponge was purchased from Jiangxi Xiangen Medical Technology Development Co., Ltd. (China). *Staphylococcus aureus* (*S. aureus*, ATCC 25923), *Escherichia coli* (*E. coli*, ATCC 25922), and methicillin-resistant *Staphylococcus aureus* (*MRSA*, ATCC 43300) were obtained from the department of laboratory medicine, Guangdong Provincial People's Hospital (China). Dulbecco's modified Eagle's medium (DMEM), fetal bovine serum (FBS), and penicillin-streptomycin solution were provided by Gibco BRL (USA). The cell counting kit-8 (CCK-8) was purchased from Labgic Technology Co., Ltd. (China). The live/dead cell viability kit (Calcein-AM/PI) was purchased from Shanghai Xinyu Biotechnology Co., Ltd. (China). The Actin-Tracker Green-488 was purchased from Beyotime Biotech Inc. (China). Standard fibroblast cell line L929 fibroblasts and mouse-derived macrophages (Raw264.7) were provided by Jiangsu Keygen Biotech Co., Ltd. (China). The rabbit blood was purchased from Guangzhou Hongquan Biotechnology Co., Ltd. (China). Rabbit polyclonal antibody against CD206 and Cy3-labeled goat anti-rabbit IgG were purchased from Wuhan Servicebio Technology Co., Ltd. (China). PrimeScript™ RT Master Mix (Perfect Real Time) and TB Green® Premix Ex Taq™ (Tli RNaseH Plus) were purchased from Takara Biomedical Technology (Beijing) Co., Ltd. (China). Glyceraldehde 3-phosphate dehydrogenase (GAPDH) and all the Primers were obtained from Sangon Biotech Co., Ltd. (China). All solvents and chemicals were purchased from commercial sources and used as received, unless otherwise noted. The male Sprague Dawley (SD) rats (200-250 g) were purchased from laboratory animal center of Sun Yat-sen University (China). All rats were fed and tested in accordance

with laboratory rules and guidelines. Histological analysis was tested by Wuhan Servicebio Technology Co., Ltd. (China).

2. Preparation of Emulsion

First, ATP and T-20 were added to different weights of deionized water and stirred at 3000 rpm for 2 min to disperse uniformly. Next, different weights of WEO were added to the dispersion liquid and stirred at 11,000 rpm for 5 min to form an emulsion. The preparation parameters of emulsions are listed in **Table S3**.

3. Synthesis of Hydrogel Dressing (HD)

HD, HD-WEO, and HD-WEO-10%-T were prepared as follows: GelMA (0.2 g) was first dissolved in deionized water, then ATP and T-20 were added and stirred at 3000 rpm for 2 min to disperse uniformly. Different weights of WEO were then added and mixed at 11,000 rpm for 5 min. After that, AM (3 g), AAc-NHS (0.1 g), and APS (0.5 g) were added and mixed at 400 rpm for 1 min. Subsequently, the reaction mixture was sealed in a mold and placed in an oven at 65 °C for 150 min to form the hydrogel dressing (HD) without or with WEO (HD-WEO).

HD-WEO-10%-M was prepared as follows: GelMA (0.2 g) was first dissolved in 9 g deionized water, ATP (0.25 g) and T-20 (0.2 g) were then added and stirred at 3000 rpm for 2 min to disperse uniformly. After that, AM (3 g), AAc-NHS (0.1 g), APS (0.5 g), and WEO (1 g) were added and mixed at 400 rpm for 1 min. Subsequently, the reaction mixture was sealed in a mold and placed in an oven at 65 °C for 150 min to form the HD-WEO-10%-M. The preparation parameters of HD are listed in **Table S4**.

4. Characterization

Digital photos of the emulsions were taken with a smartphone after the emulsions were freshly prepared and stored for 48 h to evaluate the stability of the emulsions. CLSM image of PE-10% was obtained using an inverted fluorescence microscope (MF53-N+MSX2-C) to observe the distribution of ATP (stained with safranin T). The microtopography of the RDHD (HD-WEO-10% after removing oil and drying) was characterized by SEM (ZEISS Sigma 300). FT-IR spectra were recorded using a FT-IR spectrometer (Nexus 670) in the 4000-500 cm^{-1} range at room temperature. HD, HD-WEO-5%, HD-WEO-10%, and HD-WEO-10%-T were prepared with rectangular

pieces (length: 18 mm, width: 7 mm, thickness: 3 mm) for the tensile test. The tensile properties were measured by placing the HDs on the sample stage of a universal testing machine (WD-5A) with a 50 N load cell and an extension speed of 30 mm min^{-1}. The adhesion performance of HD-WEO-10% was demonstrated by application to human skin. The biocompatibility of HD-WEO-10% with cells was validated prior to the studies. Human subjects gave their complete informed consent to participate in these studies.

5. Anti-reflux test

HD-WEO-10% and HD-WEO-10%-T were saturated with Rhodamine B solution and placed on a piece of filter paper. After 30 seconds, they were removed, photographed, and observed to determine whether reflux of the Rhodamine B solution occurred at the bottom of the specimens. Subsequently, the samples were placed on a new piece of filter paper and removed after 60 seconds to be photographed and observed for a second time.

6. Swelling ratio of HD-WEO

The gravimetric method was used to determine the swelling ratio (*SR*) of the HDs at 37 °C in order to assess their effusion absorbency. A specific weight (W_0) of dressing was soaked in PBS buffer (pH 7.4) and shaken at 80 rpm at 37 °C. The weight of the swollen dressing was then measured at various time intervals (W_t). The *SR* was calculated as follows:

$$SR = \frac{W_t - W_0}{W_0} \times 100\% \qquad (1)$$

7. Antibacterial properties of HD-WEO

To investigate the antibacterial activity of the HD-WEO, tests were carried out using *S. aureus*, *E. coli*, and *MRSA*. All dressings were exposed to UV for 1 h before bacterial incubation. *S. aureus*, *E. coli*, and *MRSA* were inoculated in Luria-Bertani (LB) medium and shaken continuously at 37 °C for 24 h. Bacterial solutions (10^5 CFU mL^{-1}, 4 mL) were then added to the small glass vial containing HD, HD-WEO-10% (size: 10 ×10×1 mm) and incubated at 37 °C for 24 h. During incubation, the optical density at 600 nm of the above bacterial solutions was measured (Multiskan Go) at different times to plot bacterial growth curves. Meanwhile, the turbidity of the bacterial solutions after 24 h

were observed. In this assay, a bacterial solution without any treatment was used as the blank group. Each sample was replicated three times.

The antibacterial efficacy of HD and HD-WEO-10% was further evaluated using the plate count method, which determines the number of colony-forming units (CFU) on agar plates. Briefly, HD and HD-WEO-10% were placed in 48-well plates, immersed in 300 μL of *S. aureus*, *E. coli*, and *MRSA* (10^7 CFU mL^{-1}), and incubated at 37 °C for 12 h. The bacterial solution was then diluted 10^6-fold with PBS, and 20 μL of the diluted bacterial solution was inoculated onto agar plates. After 12 h (*S. aureus* and *E. coli*) and 24 h (*MRSA*), the bacterial colony forming units were photographed. The antibacterial ratio (*AR*) was calculated as follows:

$$AR = \frac{CFU\ of\ blank - CFU\ of\ HD}{CFU\ of\ blank} \times 100\% \quad (2)$$

An antibacterial ring test was performed to further determine the antibacterial ability of HD-WEO. The HD, HD-WEO-10%, HD-WEO-10%-T, and HD-WEO-10%-M were cut into discs (10 mm diameter) and then placed on agar plates with a bacterial solution (10^8 CFU mL^{-1}). HD-WEO-10%-T represents a hydrogel containing WEO that is prepared using T-20 stabilized emulsion. HD-WEO-10%-D refers to HD with WEO droplets present on the surface, while HD-WEO-10%-M is obtained by mixing the WEO into the hydrogel precursor prior to polymerization.

8. Biocompatibility of HD-WEO

Cell cytotoxicity and proliferation were measured using the cell counting kit-8 reagent (CCK-8). HD and HD-WEO-10% were sterilized by UV irradiation for 1 h and added in the complete medium at 0.1 g mL^{-1} for 24 h at 37 °C. L929 cells were cultured at 37 °C in DMEM supplemented with 10% FBS and 1% penicillin-streptomycin in a 5% CO_2 atmosphere at 37 °C for 24 h. After L929 fibroblasts adhered to the plate, the medium was removed and extraction solutions of HD and HD-WEO-10% were added. After 24 h, 10 μL of CCK-8 was added and stored in the dark for 1 h. The optical density (OD) of three parallel wells was then measured at 450 nm using a full-wavelength microplate reader (Multiskan GO).

Live/dead staining kits were used to stain L929 cells. Live cells were stained with

calcein-AM with green fluorescence and dead cells were stained with PI with red fluorescence. The cytoskeleton staining procedure is described below. L929 cells were cultured at a density of 10^5 mL^{-1} in 24-well plates and incubated for 24 h. The medium was then replaced with extraction solution and incubated for 24 h. Subsequently, L929 cells were fixed with 4% formaldehyde PBS at room temperature for 10 min. L929 cells were then washed three times with PBS for 10 min, permeabilized with 0.1% Triton X-100 solution for 5 min, and washed three times with PBS. Actin-Tracker Green working solution (200 μL) was then added to cover the cells in the plates, incubated at room temperature and stored in the dark for 30 min, and then washed with PBS. Finally, DAPI solution (200 μL) was used to re-stain the nuclei. Images were captured under a fluorescence microscope (ZEISS Axio Observer Z1).

A hemolytic activity assay was performed to assess the hemocompatibility of HD and HD-WEO-10%. Briefly, rabbit blood was collected and centrifuged at 4 °C for 5 min at 1500 rpm. The resulting erythrocytes were then washed with PBS and centrifuged 3 times to obtain purified erythrocytes, and then a 5% (v/v) erythrocyte-PBS solution was prepared. The extraction solutions of HD and HD-WEO-10% (500 μL) were then each mixed with 500 μL erythrocyte suspension (5% v/v) in the tube for 1 h at 37 °C. All samples were then centrifuged at 1500 rpm for 5 min, and the supernatant was collected to measure the absorbance at 540 nm using a microplate reader (Multiskan Go). A negative control (PBS) and a positive control (0.1% Triton X-100) were also included in the experiment to assess the degree of hemolysis. The hemolysis ratio (*HR*) was calculated as follows:

$$HR = \frac{OD\ of\ HD - OD\ of\ negative\ control}{OD\ of\ positive\ control - OD\ of\ negative\ control} \times 100\% \tag{3}$$

Histocompatibility test was performed by subcutaneously implanting the dressing on the dorsal side of the rat. All samples were cut into circles (diameter: 10 mm, height: 2 mm) and sterilized by UV irradiation for 1 h. Male Sprague-Dawley (SD) rats weighing 200-250 g were selected and anaesthetized. Each rat's back was shaved and sterilized with a 75% ethanol solution before a small incision was made in the symmetrical parts of the spine on the left and right sides. The subcutaneous tissue of the incision was filled

with a gelatin sponge (GS) and HD-WEO-10% before the skin was sutured. On the 7th day after surgery, the animals were euthanized, and the back tissues were collected and fixed in 4% paraformaldehyde solution for immunohistochemical staining of CD68, IL-1β, and IL-6.

9. In Vitro Macrophage Phenotype Regulation of HD-WEO

We assessed the effect of HD-WEO on macrophage polarization by examining the expression of the M2 surface marker (CD206) using immunofluorescence staining. Raw 264.7 cells were inoculated into 6-well plates at a density of 10^5 per well and cultured in DMEM medium supplemented with 10% FBS at 37 °C. The cells were then stimulated with 100 ng mL^{-1} LPS for 24 h to induce M1 polarization. Subsequently, different samples were added to the 6-well plates and incubated for 48 h. Raw 264.7 cells were then fixed with 4% paraformaldehyde, permeabilized with 0.1% Triton X-100, and blocked with 1% BSA at room temperature. RAW 264.7 cells were then incubated with rabbit polyclonal antibody against CD206 (1:150) overnight at 4°C, followed by incubation with Cy3-labeled goat anti-rabbit IgG secondary antibody (1:200) for 1 h in the dark. Finally, the nuclei were stained with DAPI and observed under a fluorescence microscope (Nikon Ti-S).

The expression levels of M1 macrophage-related genes, including iNOS, CD86, and IL-1β, and M2 macrophage-related genes including ARG-1, CD206, and IL-10, were detected by quantitative reverse transcription-PCR (RT-qPCR) after induction of macrophage polarization. Total RNA was extracted from macrophages using the RNA Rapid Extraction Kit (RN001), and reverse transcription was performed using the PrimeScript™ RT Master Mix (Perfect Real Time) (RR036A) kit and a PCR instrument (TAavanced Twin48G). The PCR reaction solution containing TB Green Premix Ex Taq II, forward primers, reverse primers, and cDNAs was then configured, followed by quantitative detection using a real-time PCR system (qTOWER^{3}G). The primer sequences are displayed in **Table S5**. The target gene is accurately quantified by detecting the intensity of the fluorescent signal in the reaction solution. GAPDH was set as the reference gene. Relative mRNA expression levels of target genes were normalized to control samples and calculated using the $2^{-\Delta\Delta Ct}$ method.

10. In Vivo Diabetic Wound Healing

Type 1 diabetic models were established using male Sprague-Dawley (SD) rats weighing between 200-250 g. Successful establishment of diabetic rats was defined as a blood glucose level of ≥ 16.7 mmol L^{-1} after one week of intraperitoneal injection of streptozotocin (STZ) solution at a dose of 60 mg kg^{-1}. The diabetic rats were first injected intraperitoneally with 1% sodium pentobarbital (50 mg kg^{-1}), depilated and sterilized with a 75% ethanol solution. Full-thickness skin wounds were made on the back of the rats using a 10 mm round punch, then 100 μL MRSA (10^8 CFU mL^{-1}) was added and cultured for 1 day to establish a rat infected diabetic wound. The wounds were then covered with gauze (GZ), gelatin sponge (GS), and HD-WEO-10%. The wounds were photographed, the wound area ratio was measured using ImageJ and calculated as follows:

$$Wound\ area\ ratio = \frac{A_{(3,7,14)}}{A_0} \times 100\% \qquad (4)$$

On the 7th and 14th days, the rats were euthanized, and the wound tissues were collected and fixed in 4% formaldehyde for histological and immunofluorescence analysis. Hematoxylin and eosin (H&E) staining was used to measure wound healing. Masson staining was used to assess collagen deposition. Immunohistochemical staining for CD31 was performed at different times to assess angiogenesis. Immunohistochemical staining for IL-1β, IL-10, CD68, CD206, and CD163 was also performed on 7th day after surgery to assess macrophage differentiation at the wound site. The results of the staining were quantified using Image J software.

Statistical analysis: Results were expressed as mean ± standard deviation (SD) of at least three independent experiments. Statistical analysis was evaluated by SPSS software (version 26.0 for Windows; SPSS, Chicago, IL, USA). Statistical differences between groups were determined by Student's t-test or one-way analysis of variance (ANOVA). Significance levels were indicated as $*p < 0.05$, $**p < 0.01$, $***p < 0.001$, and $****p < 0.0001$.

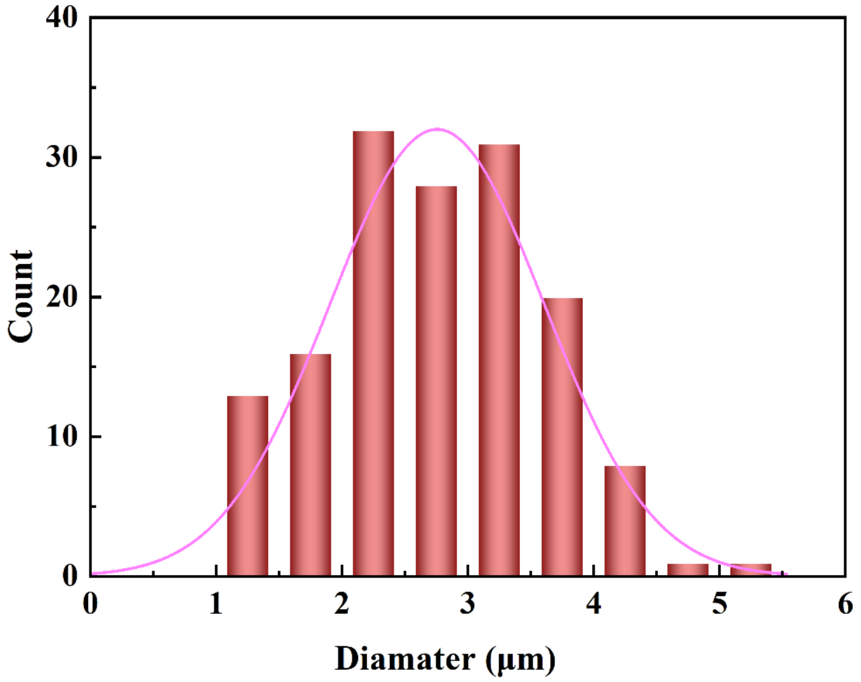

Figure S1 Pore diameter distribution of RDHD.

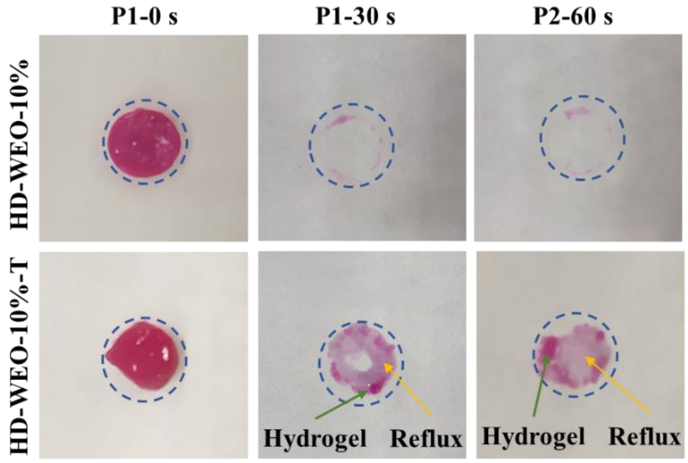

Figure S2 The anti-reflux performance of HD-WEO-10% and HD-WEO-10%-T. P1-0 s: HDs are placed directly on the first filter paper; P1-30 s: HDs are placed on the first filter paper for 30 s; P2-60 s: HDs are placed on the second filter paper for 60 s.

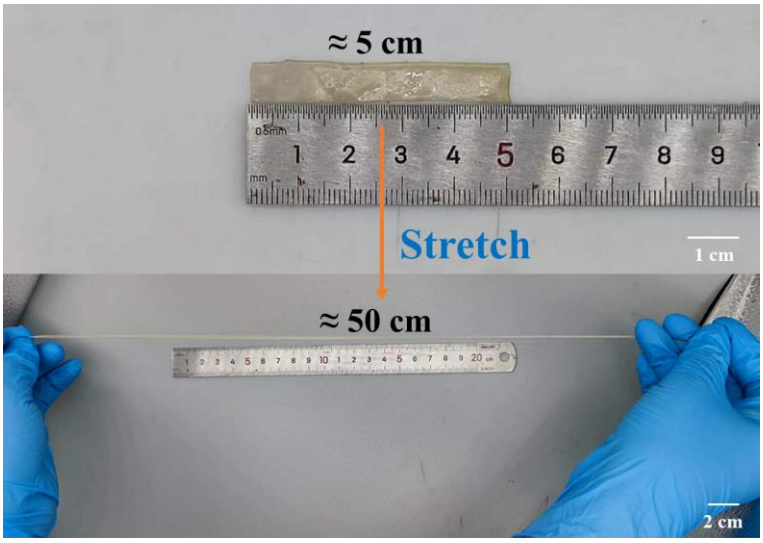

Figure S3 Digital photos of HD-WEO-10% before and after stretching.

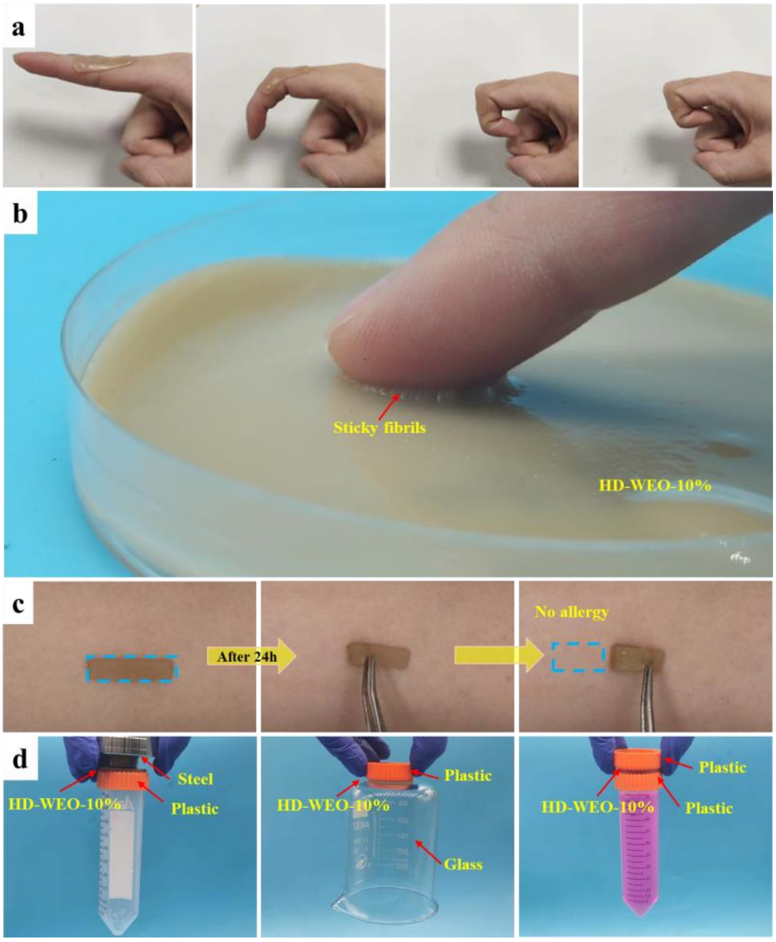

Figure S4 a) Digital photos of HD-WEO-10% adhered to finger with dynamic stretching along with the movement of joints. b) Digital photo of sticky fibrils between HD-WEO-10% and human finger. c) Digital photos of human skin exposed to HD-WEO-10% for 24 h. d) Digital photos of HD-WEO-10% adhered to various materials.

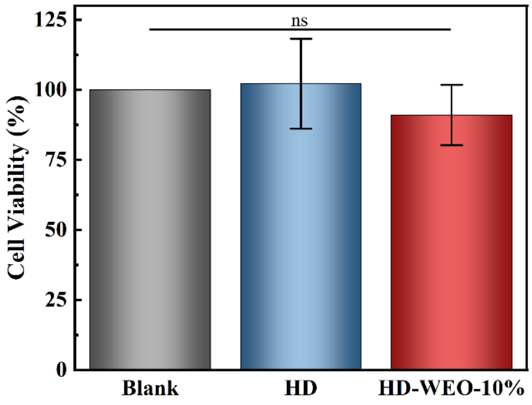

Figure S5 Cell viability of L929 cells after co-culture with the HD, HD-WEO-5% and HD-WEO-10% for 24 h. The error bars show a standard deviation (n = 3). ns: not significant.

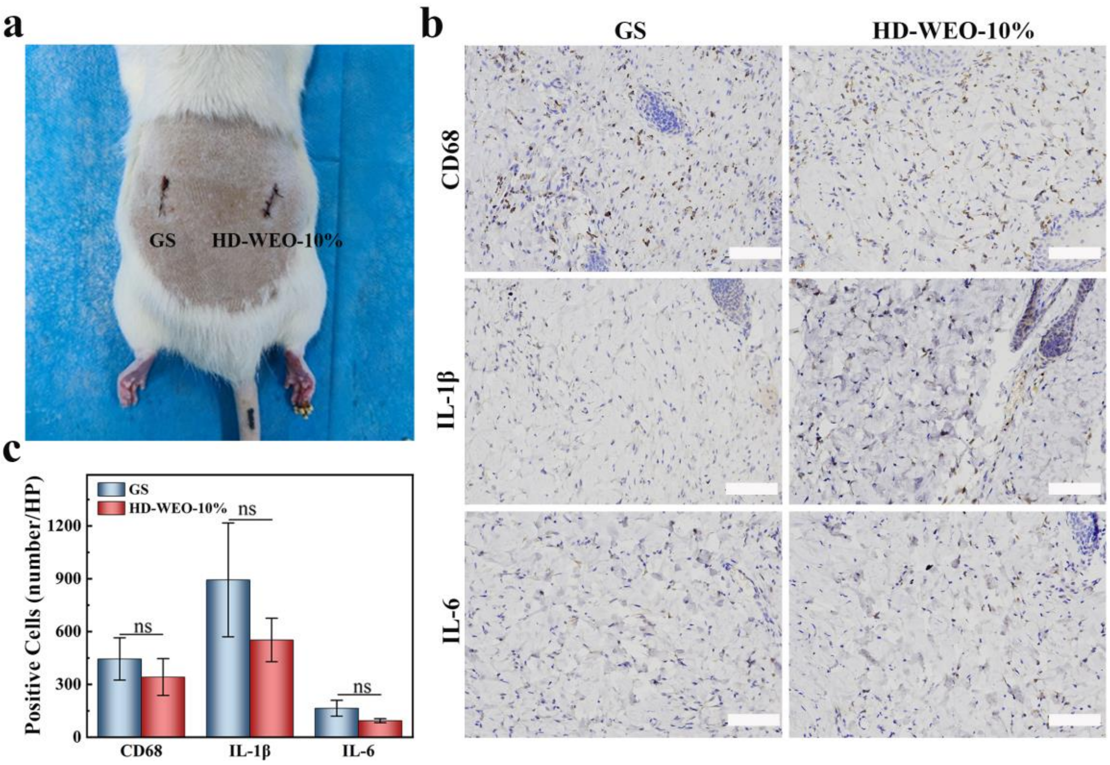

Figure S6 a) Digital photo of subcutaneous embedding experiment of the GS and HD-WEO-10% groups. b) Immunohistochemical staining images of CD68, IL-1β, and IL-6 at the subcutaneous tissue in the GS and HD-WEO-10% groups (scale bar: 100 μm). c) Statistical data of CD68, IL-1β, and IL-6 positive cells per high-power field in the GS and HD-WEO-10% groups. The error bars show a standard deviation (n = 3). ns: not significant.

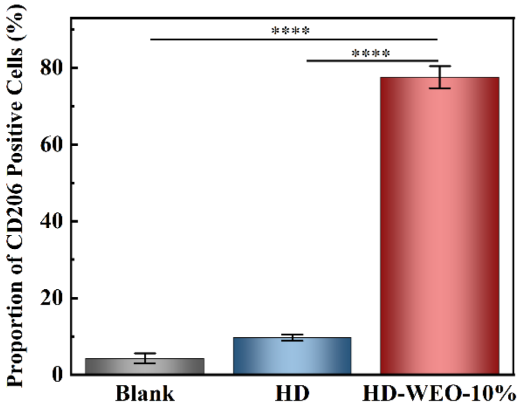

Figure S7 Proportion of CD206 positive cells per field in the blank, HD, and HD-WEO-10% groups. The error bars show a standard deviation (n = 3). ****$p < 0.0001$.

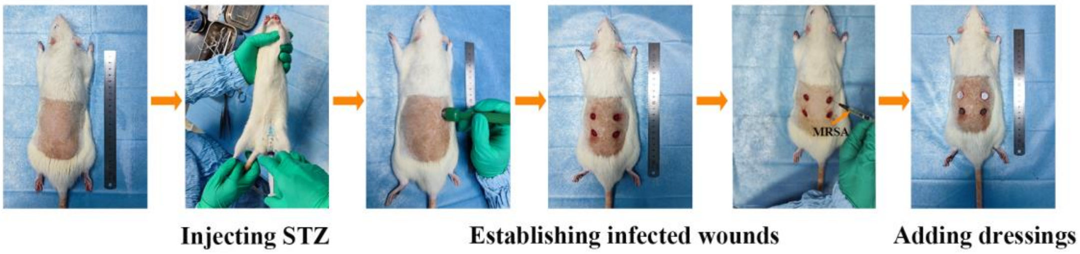

Figure S8 Surgical procedures were performed on the dorsal *MRSA*-infected diabetic wounds of rats using the GZ, GS, and HD-WEO-10% as wound dressings.

Table S1. The antibacterial agents and antibacterial ratios in the reported antibacterial hydrogel dressings

**Antibacterial strategy**	**Antibacterial agent**	**Antibacterial ratio**			**Ref.**
		S. aureus	***E. coli***	***MRSA***	
Antibiotic	Tobramycin	≈100%	≈100%	/	[1]
	Chlorhexidine acetate	≈100%	≈100%	/	[2]
	Tobramycin	≈100%	≈100%	/	[3]
Metal and metal oxides	Cu NPs and Cu^{2+}	99.90%	91%	/	[4]
	ZnO NPs	93%	92%	/	[5]
	Ag NPs	>80%	/	/	[6]
	ZnO and Zn^{2+}	80.60%	83.30%	/	[7]
Photothermal therapy	ILGA@Gel	/	/	≈100%	[8]
	rGB/QCS	/	96.60%	94.60%	[9]
	MPDA-LZM	/	≈53.22%	/	[10]
Photodynamic therapy	PCN-224(Zr/Ti) NPs	/	96.40%	96.80%	[11]
	P/M-S100	99.99%	99.99%	/	[12]
	SeV^{2+}-PT PEWD	/	/	≈80%	[13]
	PL@Pr-TCPP	≈100%	/	/	[14]
Natural plant essential oils	WEO	≈100%	≈100%	≈100%	This study

Table S2. The bacteria used in antimicrobial tests and animal experiments of reported dressings for infected diabetic wounds

Dressing	Bacteria used in antibacterial test		Bacteria used in animal experiment	Ref.
	Gram-negative	Gram-positive		
OCM@P hydrogel	*E. coli*	*S. aureus*	/	[15]
EPEG-OA@PHMS hydrogel	*P. aeruginosa*	*S. aureus*	*S. aureus*	[16]
DMN@TH/rh-EGF	*E. coli*	*S. aureus*	/	[17]
DATS@PFC&CMC	*E. coli*	*S. aureus*, *MRSA*	*MRSA*	[18]
Self-oxygenated double-layered hydrogel	*E. coli*	*S. aureus*, *MRSA*	*MRSA*	[19]
ker-PA/Fe/PP 0.5	/	*S. aureus*	/	[20]
PABC	*E. coli*	*S. aureus*	/	[21]
Met@CuPDA NPs/HG hydrogel	*E. coli*	*S. aureus, MRSA*	*S. aureus*	[22]
PDA@Ag NPs/CPHs	*E. coli*	*S. aureus*	*E. coli*, *S. aureus*	[23]
HQB	*E. coli*	*S. aureus*	/	[24]
FGMA/FG/PA	*E. coli*	*S. aureus*	*S. aureus*	[25]
ZCO-HA MN	/	*MRSA*	/	[26]
C_3N_4@Gel	*E. coli*	*S. aureus*	*S. aureus*	[27]
GOD-CP MCPS	*E. coli*	*S. aureus*	/	[28]
ACPCAH	*P. aeruginosa*	*MRSA*	*MRSA*	[29]
SF/Hb/Ga hydrogel	*P. aeruginosa*	*S. aureus*	*P. aeruginosa*	[30]
N-MOF-GO-Ag	*E. coli*, *P. aeruginosa*	*S. aureus*	/	[31]
H/C@GO-AS@bFGF	*S. aureus*	/	*S. aureus*	[32]
HD-WEO	*E. coli*	*S. aureus*, *MRSA*	*MRSA*	This study

Table S3. Preparation of Emulsion

Emulsion	H_2O	WEO	ATP	T-20
PE-5%	9.5 g	0.5 g	0.25 g	0.2 g
PE-10%	9 g	1 g	0.25 g	0.2 g
TE-10%	9 g	1 g	0 g	0.2 g

Table S4. Preparation parameters of HD

Sample	H_2O	WEO	ATP	T-20
HD	10 g	0 g	0.25 g	0.2 g
HD-WEO-5%	9.5 g	0.5 g	0.25 g	0.2 g
HD-WEO-10%	9 g	1 g	0.25 g	0.2 g
HD-WEO-10%-T	9 g	1 g	0 g	0.2 g
HD-WEO-10%-M	9 g	1 g	0.25 g	0.2 g

Table S5 Primers used in the qRT-PCR analysis

Gene	Forward primers	Reversed primers
iNOS	CCTGCTTTGTGCGAAGTGTC	CCCAAACACCAAGCTCATGC
ARG-1	ACATTGGCTTGCGAGACGTA	ATCACCTTGCCAATCCCCAG
IL-1β	TGCCACCTTTTGACAGTGATG	GTGCTGCTGCGAGATTTGAA
CD86	CTTACGGAAGCACCCACGAT	CGGCAGATATGCAGTCCCAT
IL-10	CCAAGGTGTCTACAAGGCCA	GCTCTGTCTAGGTCCTGGAGT
CD206	GTGGGGACCTGGCAAGTATCCAC	CACTGGGGTTCCATCACTCC

References

[1] C. Yang, J. Dawulieti, K. Zhang, C. Cheng, Y. Zhao, H. Hu, M. Li, M. Zhang, L. Chen, K. W. Leong, D. Shao, *Adv. Funct. Mater.* **2022**, *32*, 211169.

[2] M. Zhang, Y. Huang, W. Pan, X. Tong, Q. Zeng, T. Su, X. Qi, J. Shen, *Carbohydr. Polym.* **2021**, *253*, 117213.

[3] Y. Huang, L. Mu, X. Zhao, Y. Han, B. Guo, *ACS Nano* **2022**, *16*, 13022.

[4] X. Sun, M. Dong, Z. Guo, H. Zhang, J. Wang, P. Jia, T. Bu, Y. Liu, L. Li, L. Wang, *J. Biol. Macromol.* **2021**, *167*, 10.

[5] W. Liao, D. Yang, Z. Xu, L. Zhao, C. Mu, D. Li, L. Ge, *Adv. Healthc. Mater.* **2023**, *12*, 2203054.

[6] C. Liu, J. Ling, L.-Y. Yang, X.-k. Ouyang, N. Wang, *Carbohydr. Polym.* **2023**, *303*, 120436.

[7] J. Cheng, H. Wang, J. Gao, X. Liu, M. Li, D. Wu, J. Liu, X. Wang, Z. Wang, P. Tang, *Adv. Healthc. Mater.* **2023**, 2300312.

[8] W. Pan, B. Wu, C. Nie, T. Luo, Z. Song, J. Lv, Y. Tan, C. Liu, M. Zhong, T. Liao, Z. Wang, G. Yi, L. Zhang, X. Liu, B. Li, J. Chen, L. Zheng, *ACS Nano* **2023**, *17*, 11253.

[9] P. Zhao, Y. Zhang, X. Chen, C. Xu, J. Guo, M. Deng, X. Qu, P. Huang, Z. Feng, J. Zhang, *Adv. Sci.* **2023**, *10*, 2206585.

[10] Y. Wang, Q. Lv, Y. Chen, L. Xu, M. Feng, Z. Xiong, J. Li, J. Ren, J. Liu, B. Liu, *Acta Pharm. Sin. B* **2023**, *13*, 284.

[11] M. Chen, Z. Long, R. Dong, L. Wang, J. Zhang, S. Li, X. Zhao, X. Hou, H. Shao, X. Jiang, *Small* **2020**, *16*, 1906240.

[12] M. He, F. Ou, Y. Wu, X. Sun, X. Chen, H. Li, D. Sun, L. Zhang, *Mater. Des.* **2020**, *194*, 108913.

[13] K. Zhou, D. Chigan, L. Xu, C. Liu, R. Ding, G. Li, Z. Zhang, D. Pei, A. Li, B. Guo, X. Yan, G. He, *Small* **2021**, *17*, e2101858.

[14] P. Ran, T. Xia, H. Zheng, F. Lei, Z. Zhang, J. Wei, X. Li, *Acta Biomater.* **2023**, *155*, 292.

[15] W. Tan, T. Long, Y. Wan, B. Li, Z. Xu, L. Zhao, C. Mu, L. Ge, D. Li, *Carbohydr. Polym.* **2023**, *312*, 120824.

[16] A. Shariati, A. Moradabadi, E. Ghaznavi-Rad, M. Dadmanesh, M. Komijani, F. Nojoomi, *J. Glob. Antimicrob. Resist.* **2021**, *20*, 40.
[17] W. Liu, X. Zhai, X. Zhao, Y. Cai, X. Zhang, K. Xu, J. Weng, J. Li, X. Chen, *Adv. Healthc. Mater.* **2023**, 12, 23002.
[18] J. Chen, Z. Mu, D. Chen, C. Huang, T. Jin, L. Li, Y. Zeng, Q. Zhou, Y. Zhang, H. Mao, H. Deng, X. Shen, H. Yang, X. Cai, *Chem. Eng. J.* **2023**, *469*, 143985.
[19] Z. Zhu, L. Wang, Y. Peng, X. Chu, L. Zhou, Y. Jin, H. Guo, Q. Gao, J. Yang, X. Wang, Z. Long, Y. Ge, S. Lu, B. Wang, *Adv. Funct. Mater.* **2022**, *32*, 2201875.
[20] T. Shi, H. Lu, J. Zhu, X. Zhou, C. He, F. Li, G. Yang, *Compos. B. Eng.* **2023**, *257*, 110687.
[21] Y. Li, T. Xu, Z. Tu, W. Dai, Y. Xue, C. Tang, W. Gao, C. Mao, B. Lei, C. Lin, *Theranostics* **2020**, *10*, 4929.
[22] S. Zhu, B. Zhao, M. Li, H. Wang, J. Zhu, Q. Li, H. Gao, Q. Feng, X. Cao, *Bioact. Mater.* **2023**, *26*, 306.
[23] Y. Zhao, Z. Li, S. Song, K. Yang, H. Liu, Z. Yang, J. Wang, B. Yang, Q. Lin, *Adv. Funct. Mater.* **2019**, *29*, 1901474.
[24] S. Zhu, Q. Dai, L. Yao, Z. Wang, Z. He, M. Li, H. Wang, Q. Li, H. Gao, X. Cao, *Compos. B. Eng.* **2022**, *231*, 109569.
[25] Y. J. Fu, Y. F. Shi, L. Y. Wang, Y. F. Zhao, R. K. Wang, K. Li, S. T. Zhang, X. J. Zha, W. Wang, X. Zhao, W. Yang, *Adv. Sci.* **2023**, *10*, 2206771.
[26] J. Yang, Z. Chu, Y. Jiang, W. Zheng, J. Sun, L. Xu, Y. Ma, W. Wang, M. Shao, H. Qian, *Adv. Healthc. Mater.* **2023**, 2300725.
[27] Q. Xu, S. Chen, L. Jiang, C. Xia, L. Zeng, X. Cai, Z. Jin, S. Qin, W. Ding, Q. He, *Natl. Sci. Rev.* **2023**, *10*, nwad063.
[28] L. Wang, G. Chen, L. Fan, H. Chen, Y. Zhao, L. Lu, L. Shang., *Adv. Sci.* **2023**, *10*, 2206900.
[29] L. Shang, Y. Yu, Y. Jiang, X. Liu, N. Sui, D. Yang, Z. Zhu, *ACS Nano* **2023**, *17*, 15962.
[30] Y. Qian, C. Xu, W. Xiong, N. Jiang, Y. Zheng, X. He, F. Ding, X. Lu, J. Shen, *Chem. Eng. J.* **2021**, *417*, 129335.
[31] M. Yin, J. Wu, M. Deng, P. Wang, G. Ji, M. Wang, C. Zhou, N. T. Blum, W. Zhang, H.

Shi, N. Jia, X. Wang, P. Huang, *ACS Nano* **2021**, *15*, 17842.

[32] S. Wu, B. Qin, X. Tang, T. Cui, S. Yin, H. Dong, Y. Liu, S. Deng, H. Zhang, G. Feng, H. Xie, L. Lei, *Chem. Eng. J.* **2023**, *466*, 143126.
